# ‘Some people talk about children as though they’re completely different’: hospital art, architecture and design for children in modern Britain

**DOI:** 10.1136/medhum-2025-013234

**Published:** 2025-04-02

**Authors:** Victoria Bates

**Affiliations:** 1 History, University of Bristol, Bristol, England, UK

**Keywords:** Child health, art in hospitals, architecture, design

## Abstract

Children’s hospitals are often thought to be special places, marked by particular attention to emotions and careful consideration of inclusive design. Photographs of children’s hospitals, or design for children within general hospitals, often showcase primary colours and playfulness. Such aesthetic qualities are, at first glance, exceptional for healthcare environments and reinforce the idea that children’s hospitals are special or unique. This article, however, reconsiders this notion of exceptionalism in two ways. First, it uses the history of modern British hospitals to show that some of these qualities—such as bright colour and playfulness—might have once been a special feature of design for children, but were qualities of some adult hospital design by the end of the twentieth century. It makes this point, further, through a collection of interviews with professionals working in hospital art, architecture and design. In so doing, it places greater emphasis on process; interviews show the general expansion of person-centred design, and indicate that it has closed the gap between design for children and adults in both process and outcome.

 In June 1976, a new hospital centre for casualty and outpatient facilities opened in Greater Manchester, UK. The hospital was designed to create a ‘relaxed, cheerful environment for patients and staff’ including through its combination of landscaping, natural wood finishes and bright colours in waiting areas. Within this ‘cheerful’ environment, *Hospital Development* magazine reported, ‘[s]pecial attention has been paid to the needs of children, who will be able to play with toys in their own waiting room, which has been attractively decorated with scenes from Disneyland’ (Anon 1976, 12). Such an idea, that children’s design required ‘special attention’, was not new in the 1970s. There was a long history of children’s hospitals treating the art, architecture and design of children’s hospitals as exceptional or ‘special’. Cartoons had long adorned the walls of children’s hospitals, in the form of tile pictures, going back to the nineteenth century. By the mid-twentieth century, when hospital design was moving away from a preference for white towards gentle pastel shades, children’s hospitals—or children’s areas of hospitals—monopolised ‘brightness’, playfulness and primary colours.

The Manchester story indicates, though, that this model of exceptionalism was subtly on the turn. In this example, in anticipation of trends that would take on momentum in the 1980s and 1990s, the wider hospital was also ‘cheerful’ and involved ‘ample use of bright, gaily-patterned wall coverings’ (Anon 1976, 12). Over subsequent decades, the models underpinning hospital design for children, young people and adults came even closer together. As Alex Mold notes, ‘[t]he notion that patients could be regarded as consumers was not a new one in Margaret Thatcher’s Britain, but it was between 1979 and 1990 that the patient-consumer moved from the shadows to centre-stage’ (Mold 2011, 509). This trend had implications for hospital design, as well as for care. Children’s hospital design increasingly aligned with a broader model of hospital design that embraced person-centredness, inclusivity, and the goal of stimulation, play, or even joy in difficult circumstances.

This article disputes the commonly held idea that children’s hospitals or paediatric design are inherently ‘exceptional’, or rather that they are more ‘exceptional’ than other patient groups. The assumption that young patients want stimulation and older ones want calm has also waned over time: the ‘Disney’ model of children’s wards has been eroded, while more playfulness has been brought into adult hospital design. It is still common to find literature, in the UK and internationally, that emphasises children’s hospitals as ‘unique’ or ‘special’ or fundamentally ‘different’ in their requirements (Casimir 2019, 305; Dalke *et al* 2006, 349, Norton-Westwood *et al* 2011, abstract; Singh *et al* 2024, abstract). However, this article draws on historical evidence and interviews with people involved in hospital design to show that the gap is—in practice—not as wide as it first appears. There is increasing recognition that *all* patients are unique in their own way, and require patient-centred design, making children’s hospital design less of an ‘exception’ and more of a model for good practice and inclusive design in broader terms.

To make this case, the discussion below first offers some context to indicate how the idea of children’s ‘exceptional’ needs has developed historically. It outlines a brief history of children’s hospital design, demonstrating that ideas such as ‘playfulness’ and ‘brightness’ gradually expanded over time to include adolescent and adult spaces. It then turns to the recent past and present day, with a focus on interviews conducted with people involved in hospital art, architecture and design, for the project ‘Sensing Spaces of Healthcare’ (Bates 2024). These interviews provide the analytical core and focus of the article and the discussion places emphasis on understanding rationale and process, rather than outcomes. This approach allows for a novel understanding of children’s hospital design, and is an important contribution to a literature that is surprisingly light on practitioners’ voices.

Taken together, the two sections explore children’s hospital art, architecture and design, in recent history and the present day, to show how the ‘special’ or ‘exceptional’ features of children’s hospitals have become more widely incorporated into the principles of hospital design. Children’s ‘exceptionalism’ or ‘specialness’ has been somewhat eroded in a design model that focuses on understanding each and every patient—or patient group, which might be divided along lines of illness, demography, neurodiversity, or other characteristics—has their own special requirements. Children’s hospitals were early adopters of many of the approaches that now underpin models of good inclusive design, but they are no longer unique in this regard.

## Design for delight: the context

Children’s hospitals, or paediatric spaces within general hospitals, have followed a different trajectory to other hospital spaces in the UK. They have always demonstrated a greater willingness to incorporate colour, stimulation, joy and play into their designs. This is particularly true for hospital interiors, in their use of colour and illustration. In Europe, bright colours have traditionally been thought the domain of animals or the young, and this fed into hospital design (see Hunt 2022). In the late Victorian period, it was fashionable to ‘brighten the plain tiled walls of children’s wards with colourful tile pictures of nursery rhymes, fairy tales and other subjects which would interest and amuse sick children’ (Greene 1987, xiii). The use of nursery rhyme imagery sought to create the atmosphere and characteristics associated with liveliness, play and ‘amusement’.

In the early twentieth century, there was a rise of white ‘hygienic’ spaces for adults and children alike, but these did not immediately displace tile pictures for children.^1^ Tiles were also viewed as hygienic, and colourful pictures and nursery rhyme tile images were still being installed in many hospitals well into the 1930s. Great Ormond Street embraced the use of all-white tiles, but this was an uneven trend (Bates 2023, 29–30). A King’s Fund ‘model hospital’ from 1932 showed that more colourful tiles were still seen as good practice for children at the time (Science Museum Group). These tile pictures were an important example of the way that children’s needs were seen as distinct from those of other patients.

Coloured tiles fell out of fashion in spaces for children and adults alike, in favour of painted murals or adhesive cartoons and other forms of colourful design viewed as more modern. However, the notion of children’s distinct needs continued and indeed strengthened over the course of the century. As Roy Kozlovsky argues, in postwar hospital architecture, children were increasingly seen as an ‘exception’, with emotional needs that varied dramatically from those of adults (Kozlovsky 2020, 121–37; see also Chettiar 2023). Examples of influential cultural products included a 1952 film called ‘A Two-Year-Old Goes to Hospital’ (shown on British television in 1961), a booklet called *Care of Children in Hospital* by the American Academy of Pediatrics that was influenced by this film (1960), the British ‘Platt Report’ on the ‘Welfare of Children in Hospital’ (1959), and the 1961 formation of a new patient group Mother Care for Children in Hospital (renamed the National Association for the Welfare of Children in Hospital) (Shore 1965, 1; Williamson 2010, 80–1). When a new Health Building Note on ‘Hospital Accommodation for Children’ was introduced in 1984, to replace one from 1964, the first page noted that ‘in the past two decades, the greater knowledge of child development and the recognition of the fundamental emotional, as well as physical, needs of children has created a more enlightened attitude to the care of children in hospital’ (Department of Health and Social Security Welsh Office 1984, 1). There was an increased focus on children’s experiences of hospital care and the particularly stressful nature of hospital for children separated from loved ones.

This context fed a renewed vigour around the subject of children in hospitals. Hospital design—including toys, playrooms, interior decorative schemes and more—offered a way to mitigate some of the issues raised by paediatricians and others. In 1955, for example, two colleagues working in child health wrote in *The Lancet* that

[m]uch more thought is required about the general design and decoration of children’s hospitals … The child likes bright colours and attractive pictures, and there is no great difficulty in supplying these … Ideally a child who is not too ill should be admitted to a playroom attached to the ward, where the sight of cheerful toys will help him to lose his tension. (Illingworth and Holt 1955, 1257)

This publication suggested that many children’s hospitals were still in need of improvement. However, in the mid-1950s they were certainly doing more than many adult-facing spaces in this regard, and much hospital design was implicitly informed by the belief that children and ‘bright colours’ went hand-in-hand, even if just in the form of toys in playrooms.

The mid-century children’s hospital echoed the British city at this time, where there was a fear of ‘brash’ colours, and the brightest colours were not found in the built environment but in the ‘ephemera of everyday life’ (Faire and McHugh 2019, 288). Colour was traditionally brought in through objects and toys, while the design could actually be rather plain. Even in the absence of formal interior design or architectural interventions, clinical staff could have an impact on hospital spaces by ordering colourful toys or play materials (eg, see Borthwick Institute 1984-98). Toys created material, colourful and visual ‘noise’, at a time when design for adults was focused instead on calm and order. The Great Ormond Street Hospital archives hold the draft of a speech given at some point soon after the publication of the Platt report on the ‘Welfare of Children in Hospital’, to the Annual Meeting of the Association of Mother Care for Children in Hospital, which spoke positively and evocatively about disorder:

In the waiting hall at Great Ormond Street it is almost as easy to be run over by children riding pedal cars and carts as it is in the street! Toys are everywhere, the room is spacious and at the end of an outpatient session the Sister in charge of the Department is happily confronted with a scene that every mother faces after a play session in the drawing room. (Archive Service 1959-77)

Many hospital playrooms were difficult to distinguish from schools throughout the late twentieth century. It is no coincidence that school design was such a significant influence on hospital design, including colour schemes, and many children’s hospitals included schools for long-stay patients (see Newcastle Regional Hospital Board 1955; Department of Health and Social Security and Welsh Office 1976, 27). Much colour in schools and hospitals alike was in primary shades, provided through objects ranging from furniture to toys, creative materials and children’s drawings on the walls.

Over time, architects and designers began increasingly to adapt the principles of playful and colourful design for different age groups. In 1974, for example, architect Joyce Bolchover published an article in *Hospital Development* about an adolescent unit at St Mary’s Hospital in Manchester where ‘very bright colours are used in small areas – a window reveal, balcony walls, day-room wall … The colour scheme does get a reaction – the local newspaper in describing the unit called it “pop”’ (Bolchover 1974, 10). In some late twentieth-century hospitals, graffiti boards were provided for teenagers (Derbyshire Record Office 1980-90). This distinction between children of different ages, or children and adolescents, was evident throughout the late twentieth century, particularly if the site or space allowed for some separation between them. For example, when a new children’s unit was opened at Hull Royal Infirmary in 1990, the Head of Children’s Services noted that ‘the colour scheme for the older children is lovely and bright, while we’ve chosen gentler pastels for the babies’ (East Riding Archives 1990, 9). Such principles were then gradually integrated into adult care, as architects and designers started to break down the child/adult binary to show that playfulness could benefit adolescents and young adults.

Towards the end of the century, playfulness started to seep into design for adults. In line with a range of trends in care (such as patient-centredness and humanisation) and design (such as postmodernism), there was generally more emphasis on creating positive atmospheres rather than just soothing ones. Owen Hatherley, in his comments about the Private Finance Initiative (PFI) architectural ‘idiom’, argues that by the end of the twentieth century a great deal of public architecture was designed ‘as if furnishing a children’s ward’ (Hatherley 2010, VIII). Hatherley is implicitly critical of this trend. However, for others, the model of a ‘children’s ward’ has marked a helpful way to break free of institutional decorative schemes. There were no adult equivalents to playgrounds or playrooms, but there was a shift away from the assumption that ‘calm’ was the only goal of adults’ therapeutic environments. In relation to the Gloucestershire Royal National Health Service (NHS) Trust, for example, arts coordinator Anne Greer wrote in the late 1990s of creating ‘a colourful, positive environment which lifts the spirits of staff, patients and visitors’ (Greer 1999, 19). She brought in vibrant colours and images, including an artwork by Richard Baldwin in vibrant blue that stated ‘Laughter is the Best Medicine’, positioned on a bright orange wall (Greer 1999, 20). Such use of bright colours to encourage joy and give permission for laughter—even in difficult circumstances—can be seen as playful.

Some patients also used language around play and fun to describe these new, more vivid colour schemes. Giving feedback on the West Dorset Hospital in the early 1990s, one patient noted that it was ‘smart’ and looked ‘rather Legoland-ish’ (Wickings 1994, 35). In architectural terms, work on US hospital design has argued that modern hospitals have been notoriously ‘conservative’, with ‘cautious’ people making the decisions (Verderber and Fine 2000, 86; Sloane and Sloane 2003, 94). However, the playful principles of postmodernism still had some influence. To quote Stephen Verderber and David J Fine,

[i]f modern buildings were, according to their critics, severe, conservative, monotonous, minimalist, and restrictive, postmodern buildings could be the opposite: unorthodox in their composition and use of materials, colorful, ironic, ornamented, and historicist. (Verderber and Fine 2000, 86)

There was some evidence of colourful and unorthodox hospital design in Britain, even if not a full-scale embrace of postmodernism. Adult and children’s hospitals alike have turned to the atrium in architecture, which is a feature of postmodernism, referencing ‘a building type outside its architecture vocabulary: the shopping mall’ (Adams and Theodore 2002, 231). Recent examples that show similar design principles in this regard are: the Alder Hey Children’s Hospital (2016), discussed further below, which also drew on playful models such as the school and the ship; and Southmead’s Brunel Building (2014). It may be significant that both examples were designed by Building Design Partnership (BDP). Although different architects led the projects (see Casey 2023; Zucchi 2023), there is evidence of shared strategic and aesthetic principles across the firm, for adults and children alike.

This is not to claim that children and adults were conflated in hospital design, or that their needs were seen as the same. A 1984 Health Building Note on ‘Hospital Accommodation for Children’ emphasised that the ‘effects of separation from home and family’ were a particular concern because children are young (Department of Health and Social Security Welsh Office 1984, 11). When a Hull Children’s Unit opened in 1990, the Health Authority’s Acting Director of Planning declared that ‘[w]e are putting the patients first and the whole concept of the design takes into account children’s needs and aspirations regarding them as special people in their own right – not just as small adults’ (East Riding Archives 1990, 19). There was, then, a recognition both that children were not just ‘small adults’ *and* that adults had not lost their need for stimulation and playfulness. The principles of person-centred design were broadened, so that *all* patients should be treated as ‘special people in their own right’. By the early 2000s, publications on hospital colour schemes emphasised that ‘children’ were a particularly important age group, but so were ‘adolescents’, ‘young adult – middle aged’ and the ‘elderly’ (Dalke and Matheson 2007, 10). These principles went beyond age, as there was also a growing recognition that ‘people of different gender[s] and from different ethnic backgrounds may have varying levels of environmental needs and expectations, and ascribe different meanings to their surroundings’ (Dalke and Matheson 2007, 10). The gap, between design for children and adults, has closed because of a greater emphasis on understanding the ‘unique’ needs of all user groups (see, eg, Fleetwood-Smith 2024 on the ‘unique’ requirements of maternity spaces).

The rise of playful design for adults brought the aesthetics of spaces for children and adults closer to one another. Delight was no longer purely the domain of the young, as there was a general turn in architecture, art, and design towards more playful postmodern aesthetics in the late twentieth century. Design for adults also increasingly incorporated other elements of what had previously made children’s hospitals ‘exceptional’, for example, inclusive design and an emphasis on including the patients’ voice—or the ‘patient-consumer’—in design decisions since the 1980s and 1990s (see Mold 2011). The idea of inviting patients to be more active participants in hospital spaces was not simply a decorative choice, but aligned with shifting healthcare principles in the NHS. Overall, though many people still describe children’s hospitals as ‘special’ or an ‘exception’, the gap between designing for children and others has gradually closed. This brief background underpins the analysis that follows, in showing a subtle tension between the common idea that children’s needs are fundamentally different from those of adults, and the recognition that hospital design for children and adults actually follows similar ‘good practice’ principles.

## The end of exceptionalism?

At first glance, children’s hospital design remains rather exceptional. Site visits, photographs and published reports reinforce the idea that these are special places that pay careful attention to the emotional experience of hospital visits. However, published reports and finished buildings can only ever tell part of a story, and the record often lacks details of the rationale and process that underpinned hospital art, architecture or design. This article offers an alternative perspective by focusing on interviews rather than images and thus shows that design for children and adults is more closely aligned—in process, principle and outcome—than might be assumed by looking at images of a few high-profile children’s hospitals.

Over the course of 2022–2023, the author spoke to 26 interviewees, recruited based on their experience in hospital art, architecture or design for the NHS. Interviewees included architects, artists, art coordinators, consultants, estates managers, object designers, interior designers, interior architects and planners. All interviewees were adults. Categorised by type and gender, there were: 12 artists, art coordinators or artist-consultants (7 female, 4 male, 1 anon); 6 architects (3 female, 3 male); 3 interior designers and interior architects (2 male, 1 female); 1 object designer (male); 3 planning or estates managers (1 female, 1 male, 1 anon); and 1 clinician (male). The subject of designing for children or paediatrics arose in interviews with 16 participants, sometimes just as a sentence or two, and sometimes as a longer discussion (eg, involving people who built or worked at children’s hospitals). These interviews were designed in line with oral history methods and included some people with long years of service or who were retired, alongside some with more recent and ongoing experience. The interviews were semi-structured and involved a range of questions, including – for example – career trajectories and design processes.

These interviews support the argument made above: that the gap between hospital art, architecture, and design for children and adults has closed significantly since the mid-twentieth century. The discussion that follows analyses three themes: methods, inclusive principles and design outcomes. It shows that many of the interviewees recognised that their work in hospital art, architecture or design for children replicated many of the general ‘good practice’ principles of hospital design. Even when interviewees spoke about the exceptional or unique requirements of children’s spaces, they would often note that these also applied to adults. In some cases, people worked across a range of different types of space, and would make similar comments in relation to their work for adults, children and young people.

Participation and consultation were repeated interview themes, in part because I specifically asked interviewees about their process and who was involved in it. Some interviewees leaned towards the idea that children were uniquely difficult patients in terms of understanding, requiring special approaches to consultation. However, they also noted that *any* hospital design should involve extensive consultation with user groups of any age. To give one example in some depth, architect Benedict Zucchi, an architect and a principal of BDP – whose work includes the high-profile Alder Hey hospital in Liverpool – spoke about his recent work in hospital design for children:

There’ll always be some kind of steering group which will have representation from different elements of the client organisation, [and] sometimes has external representatives of one kind or another. Might be the municipality occasionally, might be a patient representative. And that steering group, you know, in discussion with them over a period of months, we'll gradually sort of work our way toward something that feels like the right approach, still relatively high-level. And then we will show that to different groups, clinical groups in [the] case of children, always to some children’s forum or parent and children forum, probably to a community group … always to local planners in the borough or city to gauge their reaction and how it responds to the context and so on, scale, transport all those kind of things. And then from there we, at some point we say, “well this is the concept that everyone likes” and we work it up in more detail … sort of running across everything is the experience of moving through the hospital and the quality of that experience and the characteristics of, you know, daylight and views and gardens. (Zucchi 2023)

This description of the design process, and emphasis on the principle of ‘experience’ and the ‘quality of that experience’ is not specific to children. The hospitals that emerge might look unique, exceptional or specific to children, but the underlying process is one of understanding user needs, and would be the same for adults. This is representative of a more general shift towards collaborative, consultative and participatory approaches to artwork in recent years: to quote Jane Willis, at the time director of hospital arts consultancy Willis Newson, ‘the idea of doing *with*, not doing *to*, is critical’ (Willis 2023; see also Noble 2023).

Caroline Barnes—at the time of interview, art and design coordinator for Yeovil District Hospital—similarly emphasised the importance of consultation when working with children and young people.^2^ She gave an example of a recent project for a mental health waiting space that lacked a window, and said, ‘my first point of call is [asking] “how do you want to make it feel?”’ This echoes Zucchi’s experience-led approach to architecture, and the project went on to be a ‘co-design project … with young people’ who ‘looked at all sorts of different things over, I think six weeks. They came up with [something] … a bit manga, a bit illustrative, quite soft colours’ (Barnes 2023). Barnes went on to give an example of another space for children that had not involved consultation and which was ‘soulless’, with ‘classic sort of bright colour tub chairs and sort of early learning centre kind of plastic toys’ (Barnes 2023). This interview, therefore, at first glance, indicates the specific problems of adults assuming that they know what children want, and the particular value of consultation with young people. However, Barnes also went on to express very similar ideas, in almost identical language, for adults:

My principle is, “how do you want it to feel?” And that could be - so we've got some x-ray art in some of the rooms where you’d have a mammogram … x-rays of seed pods going round in a sort of, kind of circle and if you count them all, you know, you could count them all ten times or something like that. We worked out that actually it’s functional, it’s beautiful, it’s distraction, but it’s functional because your mammogram in them days took, I don't know, however many minutes and yes, there’s stuff like that. (Barnes 2023)

This echoed some of the decisions made for the mental health waiting room for young people, in which they ‘put in designs about little insects that you could count. So that, you know, going along this sort of little line, you know, you could just [say] “okay, let’s count to 60”’ (Barnes 2023). The underpinning principle, then, of ‘how do you want it to feel?’—and ideally asking people this question—shaped her work for children and adults alike, sometimes with similar outcomes.

Other interviewees even corrected themselves when noting that participatory or consultative methods for children were specific. Colin Riches, an artist who ran visual art projects as part of the Isle of Wight Healing Arts programme for over 20 years from the mid-1990s, noted that the arts were a particularly powerful communication tool for children: ‘that’s the great advantage of the arts, particularly with children. Because they will expose things visually [for] which they can’t find the words’. However, he went on to say that it is ‘[t]he same with adults really’ (Riches 2023). Similarly, Caroline Moore—at the time Head of Arts at Great Ormond Street Hospital—when asked what was specific about arts for children, said ‘I think there’s certainly an increased willingness to take part, maybe. Actually, no, I take that back because [pause] okay, so forget that side of things, because whenever I have engaged with any patient regardless of their age, they've always been really willing and excited to participate’ (Moore 2023).

In line with this growing emphasis on user experience, interviewees also often emphasised a need to consider diversity through more inclusive or ‘universal’ design. This trend also brings design for children and adults closer to one another, as hospitals might be inhabited by a wide range of people. It has long been recognised that families are present in children’s wards, but there has also been growing recognition of the need to design other spaces for physical and mental diversity. Children are, of course, often also present in adult wards and—to quote two NHS consultants writing on children’s playground equipment in 1993—‘not all children in hospital are ill’ (Miles and Bennett 1993, 7). While acknowledging the specific requirements of different people in hospitals, there has also been a turn towards recognising that even similar demographic, social or medical groups will incorporate diversity. Children, young people and adults are no longer treated as homogeneous groups. A clinical planner and quality and access specialist spoke to exactly this issue:

[someone might say] “well, it’s just children up there” and you think okay, but when they have just come around from an anaesthetic they need someone in the toilet with them. Also, some of our children are like adults. We don’t do things like little toilets and big toilets. We just do good, well-designed bathrooms. There is lots and lots of design out there. People are coming round to it. Architects are pretty good on it if they are worth their salt, and also furniture companies are very good. (Anon 2022)

Ellie Richardson, a Senior Healthcare Planner at Guy’s and St. Thomas’s hospital, made almost exactly the same point in her interview:

IV: So, is it very different having a child-focused role, or not? Is it the same principles?

IE: I think it’s a really good discipline to do paediatrics, because you've got to cater for the family. There’s a real heavy family focus, and you get a massive range of patient sizes as well. So in terms of equality, diversity, and inclusion, we know that adults come in different sizes, and it makes you think in that way … have we included all the possibilities for how this room will function? Will it function for a baby, will it function for a toddler, will it function for a preteen, will it function for somebody who’s an adult size? So I think it’s quite a good discipline. In terms of patient consultation, Great Ormond Street is an exemplar in that they do really good consultation, the charity is very involved as well. (Richardson 2023)

The principle underpinning these discussions, then, is that there is a need for patient-centred and user-centred design that recognises both the specificity of a user group *and* the diversity that might exist within that group. Interviewees indicate that paediatrics or designing for children is good ‘discipline’ or training in principles that should be taken on more widely, offering a model for good practice.

This issue also came up in conversations with people from other fields of work. Art coordinator Caroline Barnes similarly noted that

[g]ood design is good for everyone. So good dementia-friendly design is good for everyone. Clear pictorial signage is good for people who, you know, come in with concussion or learning disability or whatever it is or just Joe Public who might be a bit anxious because they're in a hospital. So good design is good design. It doesn't have to be specific for a particular condition. (Barnes 2003)

This idea that ‘good design is good design’ is increasingly widespread, and not necessarily in tension with the trend to think more about patients as distinct groups with specific needs or experiences. In the examples that Barnes gave – of designing for young people’s mental health and for a mammogram room – similar principles (distraction through artwork that encouraged counting) operated successfully when adapted for two very different groups.

In consequence of this careful balance between inclusive design and recognition of the specific needs of all patient groups, the design of children’s hospitals is not as distinct as it once was. There is increasing recognition that children can cope with—or even benefit from—more adult design and themes, as well as interest in making spaces more vibrant and joyful for adults. In my interview with architect Benedict Zucchi, he explicitly rejected the common assumption that design for children must be fundamentally different:

I think tuning the conversation to different groups, you - I mean, some people talk about children as though they're completely different and, you know, children are somehow fazed by big buildings. I've never thought that. I mean, not if you do it in the right way. I mean, I'm fazed by the Royal Free and I'm not a child … as a teenager looking at the Royal Free, I thought it was awful and I still do. And it’s not just because it’s big, because big can be exciting. You know, there’s nothing intrinsically wrong with big, you know the skyscraper in New York can be fabulous and children love nothing more than like anyone to get to the top of the skyscraper and look at the view. You know, it’s really exciting. The Pompidou Centre, going to the top up the escalator, I mean, I'm not great with heights, but it’s fun, you know. So I don't think there’s an absolute - there are [not] any absolutes there. (Zucchi 2023)

The idea that ‘I don’t think … there are any absolutes there’ is another key guiding principle for hospital design when it is approached with a truly open mind about the needs of patients, visitors and staff. Zucchi spoke of specific design inspiration for the BDP children’s hospitals that drew on school architecture, for example, using ‘play decks’ to reduce the feeling of scale, but also noted that in many ways the design spoke to a more universal need: the ‘feeling that everybody wants to be close to the ground in an ideal world, because, I don't know, it promotes more interaction, it feels safer, it’s where trees and plants grow, and I suppose it’s man’s natural habitat to be with your feet on the ground’ (Zucchi 2023).

Other interviewees similarly indicated that the gaps between their work for child and adult audiences were not dramatic. One artist, Sian Tucker, mentioned that she had previously worked on ‘children’s book illustration … simple shapes and bright colours and identification of things’ (Tucker 2022). Tucker’s interest in simple shapes and bright colours implicitly aligns with her most famous work, *Falling Leaves* (1993). This vibrant mobile hangs in the atrium of Chelsea and Westminster Hospital, with its bright Matisse-like leaves visible to patients, visitors, and staff of all ages. Other interviewees spoke about the importance of supporting wayfinding for children in hospitals and community spaces. Some of these measures were adapted specifically for a younger audience, such as using animals or brightly coloured prompts to support disabled children to navigate (Craft 2022). However, the use of bright colours or artworks as wayfinding devices is similarly widespread in hospital design for adults (Nicoll 1995, 24).

Some design for children and young people is also losing its ‘Disney’ edge, and there is a more mature approach. This erodes the ‘exceptional’ nature of children’s hospital design from a different direction, recognising—again—that there is neurodiversity among children and that one size does not fit all. Maria Luigia Assirelli, then a Senior Associate (now Director) at Floyd Slaski Architects, spoke to me about this issue:

There’s always been the theory that children like primary colours which is right, but I think children with neurodiversity like pastel colour[s] … I try to use pastel[s] because it’s more friendly for everyone. And, I think that sometimes it’s difficult because of course you talk with someone else and they say, “well children like colours” which is true, and you want everyone to be happy. But I think if you use the right colours then you make a very nice environment, it’s still working very well and is friendly for any categories. (Assirelli 2023).

In this interview, Assirelli noted the common assumptions about children and bright colours and resisted the temptation to homogenise or treat them as a coherent group. She recognised the specific needs of children, as well as the fact that children are a (neuro)diverse group, some of whom might appreciate muted colour schemes traditionally seen as more ‘adult’. Without entirely rejecting the commonly held idea that many children like primary colours, Assirelli showed the value of a more nuanced and inclusive approach that allows for heterogeneity in an age group.

This article opened with a much-praised example from 1976 of a hospital decorated with ‘scenes from Disneyland’, but there is now a ‘beyond Disney’ approach to children’s hospital design (see Coad and Coad 2008, 35). According to Assirelli, such complexity has been embraced more in the last decade. She noted the turn to a more multisensory approach, which goes beyond Disney wall stickers:

[U]ntil, I feel, 2010 … [people were] still sticking on the wall, you know, the Disneys, the Spiderman. Since I would say [20]15, there’s been more understanding of therapeutic art in the hospital … I think the lockdown really emphasised [a] lot of other sensory issues, like smell was never taken in[to] consideration … Now with COVID, we've got an increased need for people that can’t smell or their smell has been altered and suddenly it’s been taken in[to] consideration more deeply in the design. (Assirelli 2023)

Other interviewees similarly noted the work involved in moving away from Disney-based clichés or assumptions about child-friendly design. Penny Calvert, team coordinator of ArtCare (the arts and health service at Salisbury District Hospital), noted that

if we’d have gone with what the staff asked for a new children’s unit, we would have done a new building with stickers on the cupboards with Disney [characters] … in their head that’s the solution and that’s because that’s all that their experience is. They are professional clinicians whose superpower is healing people. They are not interior designers. But again, an interior designer might come along and then choose things … that [are] never going to withstand, you know, chlorine-based cleaning. So there’s this overlap in-between where everyone has to kind of appreciate that for this setting you've got to tweak or change or be more open or problem solve. (Calvert 2023)

Calvert’s comments also raise an important issue about the value of professional expertise and its role in driving these changes. The growing number of hospital arts organisations and full-time hospital arts coordinators in the late twentieth century (see Barclay 2015), and specialist architects working in healthcare, has supported a move away from assumptions that children just want primary colours and cartoons. As discussed, the turn to more consultation-based and participatory design approaches, with a focus on patients’ wants and needs, has also subtly shifted ideas about ‘therapeutic environments’. Rather than designing *for* patients, based on psychological research or assumptions about what was good for them (primary colours, Disney), there was a growing emphasis on designing *with* patients. This approach has resulted in greater complexity and nuance in design for children and young people—at least after infancy, when meaningful consultation is possible (Birch *et al* 2007 included participants aged between 4 years and 16 years). It has equally, though, undermined assumptions about what design is ‘good for’ adults.

The idea that children need only positive distractions, and are unable to face the emotional consequences of ill health, is also being gradually eroded. Speaking to Caroline Moore from Great Ormond Street Hospital, I asked her to expand on her use of the word ‘challenging’ to describe artwork or creative consultation with children. She responded:

There is an assumption that children can't face difficult topics, or that whilst you're in hospital you wouldn't want to engage with, you know, whatever challenging content is. And as a team, certainly it makes our jobs harder, but also that’s what’s needed. So, for instance, in the coming year we're going to be working with a palliative care team to hopefully bring together families, clinical staff, mortuary staff, all working in palliative care to - we don't know how we're doing this yet, but to do some work around that process creatively … And I think it’s our responsibility to do that, it’s our responsibility to be able to identify those challenges, and use these means to explore them, because that is where art really is a benefit. (Moore 2023)

The concept of using creativity to explore or express difficult subjects is not new, and there is a long history of art therapy grounded in work with children and young people (Hogan 2001). However, in hospitals, the goal of artwork and creativity has traditionally been distraction rather than therapy, with a focus on positive imagery. The careful move towards engaging with more ‘adult’ themes, in this example in relation to palliative care for children, is an important shift. While adults have been permitted more joy in hospitals, more space is also being created for children to engage with complex emotions such as grief and fear. Moore’s remit included both participatory arts and creative consultations that would ultimately feed into hospital design, interiors and artwork, so some of these conversations might materially reshape the hospital. It is significant, as well, that Moore explicitly spoke about working with different groups of clinical and non-clinical staff in this process. Though there has been a general turn to patient-centred care and design, staff consulted in these processes were also designing their own working environments; in the palliative care example given by Moore, these are also particularly difficult working conditions. There is nuance and complexity in recognising the ‘challenging’ nature of hospital for a range of people—children of different ages, staff and families—rather than turning directly to Disney-based distractions.

Hospital artworks for children, young people and adults have occasionally been made interchangeable for entirely practical reasons: the movement of departments. With Guy Eades, former director of the Isle of Wight NHS Trust ‘Healing Arts’ programme, I had the following exchange:

IE: … hospitals are constantly changing, re-opening, re-organising themselves. So then there was a new mental illness unit built in [19]90 and then another one in 2000. Then they added a chemotherapy unit and then they revised the children’s unit and so it was all constantly evolving.

IV: That’s an interesting point, actually, because I feel when I visited today, I noticed I was looking at some artwork and then the person I was with said, “oh yes, this used to be the children’s unit” and I think that’s interesting. Like, was this artwork designed with children in mind but now it’s no longer for children? How does that work?

IE: Well, I mean, you design it obviously for the space and the type of practice it’s doing and if - I mean, if it moves, if it’s capable of being moved, you move it but that isn't always the case. So you sometimes have to re-commission things and then possibly recycle. I mean in a case – yes, the children’s unit has moved three times, I think but then you get outliers so you know, there are other buildings on the Isle of Wight where children’s healthcare is delivered or received, so you can probably relocate certain things if they don't fit into the new area. (Eades 2023)

In some hospitals, as in the example given by Eades, artwork design for children could become artwork for adults as departments moved around. While ‘inclusive design’ was an important principle, it was also increasingly a practical one that would allow artwork for adults and children to be more interchangeable and the space to be more flexible.

It is important, of course, not to overstate the ‘end of exceptionalism’. Many interviewees did still express the sentiment that children were a particularly vulnerable group of patients, because of the emotional challenges of being separated from families if staying in hospital for a prolonged period. Moore noted that children in hospital are ‘missing out on so many really critical things that we take for granted, like being able to go on playdates or going out to a museum or gallery. So to be able to bring as much normality into the hospital as possible feels important’ (Moore 2023; for similar points see Verschoren *et al* 2015, 9). The general and enduring belief that children’s hospitals or paediatric care are special can also be a self-fulfilling prophecy that makes people more open to experimentation. As Zucchi noted:

I think there’s the other dimension of children’s hospitals or maybe any building for children is that somehow it changes the attitudes of the adults. So suddenly they become much more sort of open … [they] put themselves in the shoes of the children, and it sort of removes constraints to the imagination. They stop being serious adults who I don’t know, think within certain parameters of, you know, the money or you know, a pure function, and they think, oh no, no, no what would be best for the children, for their feelings, for the feelings of their family and their siblings and, you know, maybe even the community. It somehow opens up the conversation and it gives us more space to focus on qualitative rather than quantitative aspects. (Zucchi 2023)

This comment is crucial for understanding that—though the gap between hospital design for children and adults has narrowed in practice—it still endures as a cultural idea. This can, in itself, make the process of designing for children feel more ‘free’ and open, even though architects, artists and designers actually adhere to very similar principles of ‘experience-led’ design for all people who spend time in hospitals. This cultural idea, that there is something innately special or different about children’s hospitals, also has practical effects. For example, it shapes the funding and procurement landscape, with more resources dedicated specifically to art, architecture and design. These features are less likely to be seen as ‘nice to have’ in relation to children, making such projects more resistant to the financial pressures that can lead design visions to be compromised in other healthcare projects (see, eg, the discussion of funding, bids and ‘wow factor’ in McLaughlan and Willis 2021, 1202).

## Conclusions

In 2024, the *Healthcare Design* website gave its list of ‘10 Pediatric Healthcare Projects To Watch’ from across the world. ‘These facilities are designed with the whole family in mind’, it noted, ‘incorporating design elements such as biophilic design, positive distractions, technology, and thoughtful color palettes’ (Walker 2024). These design elements are no longer unique to children’s hospitals and are widespread principles in hospital design. Design for adults, young people and children alike is increasingly shaped by similar ideas: experience and person-led design, which recognises the individual needs of different groups, but is also inclusive and caters for diversity within them. Common principles of ‘healing places’ are often applied across both, including biophilia, distraction and ‘thoughtful’ colour (Gesler 2003). Careful consideration is given to age, including how it intersects with other issues such as the nature of illness, and children are no longer treated as a homogeneous group.

Historical context, provided at the start of this analysis, helps to understand some of this story. This article has outlined some of the ways and reasons why the gap between children’s and adults’ hospital design has closed, particularly with the broader rise of the ‘patient-consumer’ and patient-centred design since the 1980s. It also, though, showed how and why the idea of children as a distinct and particularly significant group became so powerful. This cultural model of children as ‘special’ in relation to hospital design still endures. Children’s hospital design is, of course, in some ways distinct; for example, design for children opens up more playful and experimental opportunities precisely because of the social and cultural expectations around it. However, interviews with expert practitioners show that the difference is not so dramatic in practice. The model of ‘how do you want it to feel?’ is applied widely, to draw out the distinct and unique qualities of a *range* of different patient groups. Children are perhaps now not ‘exceptional’, but one of many different, complex and constantly evolving groups for which hospital art, architecture and design need to be adapted.

This article has focused on qualitative interviews, rather than on literature or case studies. It has done so to attend to practice and principles, rather than products or outcomes. This approach nuances some of the assumptions that might be made about the exceptional nature of design for children’s hospitals, based on images, site visits or publications alone. Conversations with professionals who work with children, young people and adults alike show that similar principles inform their work for all groups—even when outcomes might look dramatically different. That said, outcomes are also not as different as they once were; professionals increasingly listen carefully to all patient groups, which includes acknowledging that there might be mature children (in body and mind) or playful adults, all of whom need to coexist in hospital spaces. These interviews reveal the complexities of decision-making. It is time that these trends are more widely recognised, so that we can—to return to a quote from the interview with Benedict Zucchi—go beyond ‘absolutes’ when considering children in hospital.

## Data Availability

Data are available in a public, open access repository.

## Footnotes

This section is adapted from the chapter ‘Red: Risk and reward’ in *Feeling blue: Colour and the modern British hospital* (MUP, forthcoming).

Now Creative Health Coordinator for Somerset NHS Foundation Trust.
